# Research of childhood tuberculosis in suspected populations by molecular methods: A multicenter study in China

**DOI:** 10.3389/fcimb.2022.1018699

**Published:** 2022-10-19

**Authors:** Chunling Li, Shifu Wang, Hui Yu, Jiangxia Wang, Jikui Deng, Hongmei Wang, Chunzhen Hua, Zhiqiang Zhuo, Lei Chen, Jianhua Hao, Wei Gao, Hong Zhang, Ting Zhang, Hongmei Xu, Chuanqing Wang

**Affiliations:** ^1^ Clinical Microbiology Laboratory, Children’s Hospital of Fudan University, Children’s National Medical Center, Shanghai, China; ^2^ Shandong Provincial Clinical Research Center for Children's Health and Disease, Children’s Hospital Affiliated to Shandong University, Jinan, Shandong Province, China; ^3^ Division of Infectious Diseases, Children’s Hospital of Fudan University, Children’s National Medical Center, Shanghai, China; ^4^ Department of Infection Diseases Children's Hospital of Chongqing Medical University, Chongqing, China; ^5^ Department of Infectious Diseases, Shenzhen Children's Hospital, Shenzhen, Guangdong Province, China; ^6^ Division of Infectious Diseases, The Children’s Hospital, Zhejiang University School of Medicine, Hangzhou, Zhejiang Province, China; ^7^ Department of Infectious Diseases, Xiamen Children’s Hospital (Children’s Hospital of Fudan University Xiamen Branch), Xiamen, Fujian Province, China; ^8^ Department of Medical Laboratory Diagnosis Center, Xiamen Children’s Hospital (Children's Hospital of Fudan University Xiamen Branch), Xiamen, Fujian Province, China; ^9^ Department of Medical Laboratory Diagnosis Center, Children’s Hospital of Kaifeng City, Kaifeng, Henan Province, China; ^10^ Department of Infectious Diseases, Children’s Hospital of Kaifeng City, Kaifeng, Henan Province, China; ^11^ Department of Medical Laboratory Diagnosis Center, Children’s Hospital of Shanghai Jiaotong University School of Medicine, Shanghai, China; ^12^ Institue of Pediatric Infection, Immunity and Critical Care Medicine, Shanghai Children’s Hospital, Shanghai Jiao Tong University School of Medicine, Shanghai, China

**Keywords:** children, pulmonary tuberculosis, extrapulmonary tuberculosis, CPA, molecular

## Abstract

The research of childhood tuberculosis is inadequate in china. The cross-priming amplification (CPA) of specific DNA in clinical samples is increasingly adopted for the diagnosis of childhood tuberculosis. In this study, a multicenter research was performed to investigate the incidence and characteristics of childhood tuberculosis in suspected populations mainly by CPA method. 851 children suspected of tuberculosis were enrolled in seven centers across China. All samples were tested by a CPA method and 159 subjects were tested by Xpert MTB/RIF and liquid culture method in parallel to assess the reliability of the CPA method. A positive result in any one of the three methods provided a definitive diagnosis of *Mycobacterium tuberculosis complex* (MTBC) infection. The MTBC-positive rate was 9.5% (81/851) by the combined methods; 93.8% of the cases were detected by CPA technology (76/81). The rate of pulmonary infection was significantly higher than that of extrapulmonary infection (7.1%, 60/851 vs 2.5%, 21/851; P < 0.001). Scrofula was the predominant type of extrapulmonary tuberculosis. The MTBC positive rates in 12-18-year-old group (middle school), was 28.4% (23/81), higher than in those under-six-year-old (preschool; 39/525) and the 6~11-year-old (primary school; 18/235) groups combined (P < 0.001). The MTBC positive rate in patients with a clear history of tuberculosis exposure was significantly higher than in cases in which there was no history of tuberculosis contact(35.3%, 18/51 vs 7.8%, 61/782; P < 0.001). In conclusion, this multicenter investigation showed that pulmonary tuberculosis and extrapulmonary tuberculosis are not uncommon in children in China, with teenagers being particularly susceptible to infection. The incidence of pulmonary tuberculosis in children is higher than that of extrapulmonary tuberculosis. History of exposure to tuberculosis is a high risk factor for childhood tuberculosis.

## Introduction

Childhood tuberculosis is still a major public health problem in the world. There are at least one million new cases of tuberculosis in children every year, and about 200,000 children died of tuberculosis in 2021 (Nelson and Wells, 2004; Xu et al., 2012; Murray et al., 2014; WHO, 2022) Children are vulnerable to MTBC due to their immature immune systems. Children younger than three years old are more likely to develop severe tuberculosis, such as TB meningitis and disseminated tuberculosis, which threaten their health and lives. (Swaminathan and Rekha, 2010; Reyes et al., 2020; Thadchanamoorthy and Dayasiri, 2020) The incidence of tuberculosis in children and adolescents has risen considerably in recent years (Marais et al., 2004; Dodd et al., 2016; Seddon et al., 2018; Horton et al., 2020) and an annual increase of about 99,000 new cases of tuberculosis in children under the age of 14 in China has been reported, (Small and Pai, 2010) but the new tuberculosis cases were most likely underestimated compared to the reports from other high-tuberculosis-burden countries, childhood tuberculosis deserves more attention in China.

Low bacterial load and lack of specific clinical manifestations make the tuberculosis diagnosis difficult in pediatric patients. The tuberculin skin test (TST) has been used as a rapid and easy tool for tuberculosis screening, but it suffers from poor specificity due to a cross-reaction after Bacillus Calmette-Guerin (BCG) vaccination or infection with non-tuberculosis mycobacteria (NTM). Moreover, the traditional laboratory methods have their drawbacks in detecting tuberculosis. For example, smear microscopy suffers from poor sensitivity, and the culture of bacterial colonies on solid media, although potentially with high sensitivity and specificity, is time-consuming (about 6~8 weeks). (Small and Pai, 2010; Zheng et al., 2022) These drawbacks often lead to failure or delay in childhood tuberculosis diagnosis. In recent years, molecular diagnostic techniques have become widely used in the diagnosis and epidemiological investigation of infection by bacteria of the mycobacterium tuberculosis complex (MTBC). (Carpentier et al., 1995; Detjen et al., 2015; Aurilio et al., 2020) The Xpert MTB/RIF method is recommended by the WHO for rapid diagnosis of TB, replacing the traditional time-consuming culture method.

CPA is is a new nucleic acid isothermal amplification method performed by chain replacement DNA polymerase and without the need for the addition of notch enzymes or initial denaturation steps. At a constant temperature 63°C, a DNA target sequence can be amplified exponential using multiple cross-linked primers, highly specific and highly sensitive. (Xu et al., 2012) Compared with traditional PCR technology, CPA method is simple to operate and does not require PCR amplification, and is much cheaper than Xpert MTB/RIF, which is very suitable for primary hospitals in countries with a high burden of tuberculosis.

CPA technology has been certificated for clinical use in China for MTBC detection and it has been mentioned several times by WHO for TB diagnosis, in the Global Tuberculosis Annual Report, since 2019. (Ou et al., 2014; Wang et al., 2018) CPA technology has been used for rapid diagnosis of many infectious diseases, such as babesia bovis infection, scale drop disease virus infetion, avian leukosis virus subgroup J infection, african swine fever virus and feline herpesvirus type 1 (FHV-1) infecton, Bacillus cereus infection, COVID-19 infetion and so on. (Woźniakowski et al., 2018; Tan et al., 2020; Zhai et al., 2020; Prasitporn et al., 2021; Wang et al., 2021; Wu et al., 2021; Xiang et al., 2021) To ensure sensitivity and specificity, it targets the multiple-copy gene IS6110 of MTBC and is performed at a constant temperature 63°C. It has been reported that CPA can be used to diagnose pulmonary tuberculosis and peripheral tuberculosis. (Ou et al., 2014; Zhang et al., 2021; Quan et al., 2022) It may be more economical than Xpert MTB/RIF, but few children have been included in published studies.

We conducted this prospective study to investigate the incidence and characteristics of childhood tuberculosis in suspected populations mainly by CPA method in seven cities in China.

## Materials and methods

### Study setting

Between January 2019 and July 2020, eight children’s hospitals in 7 Chinese cities (Shanghai, Hangzhou, Jinan, Kaifeng, Chongqing, Xiamen, Shenzhen) participated, including Children’s Hospital of Fudan University, Children’s Hospital of Chongqing Medical University, Children's Hospital Affiliated to Shandong University, Shenzhen Children’s Hospital, Children’s Hospital of Zhejiang University, Xiamen Children’s Hospital, Children’s Hospital of Kaifeng, and the Children’s Hospital of Shanghai Jiaotong University. The Ethics Committee of Fudan University approved the investigation protocol.

### Subjects and specimens

Children presenting as outpatients and in-patients with clinically suspected tuberculosis initial consultation were enrolled. Signs and symptoms included fever, weight loss or poor weight gain, growth delay, cough, night sweats, chills, fever with neurologic symptoms, lymph node swelling, and radiographic manifestations. (Lamb and Starke, 2017; Thomas, 2017) Specimens of sputum (SP), gastric aspirate (GA), bronchial lavage fluid (BL), cerebrospinal fluid (CSF), and puncture fluid specimens (PF) were collected for MTBC detection.

### Culture

Culture was variously performed using L-J solid medium, mycobacteria growth indicator tubes (MGIT), and the Bactec 320 instrument (Becton, Dickinson, Sparks, MD, USA).

### DNA detection

#### Sample pretreatment

GA samples were stood at room temperature for 15 min to precipitate food residues and mucus. The supernatant (2~5mL) was mixed with an equal volume of 4% NaOH and shaken vigorously or vortexed for 30 s then placed at room temperature until fully digested (roughly 20 to 30 min). SP, BL, PF, and CSF samples (1~5ml) were mixed with 1 to 2 times the volume of 4% NaOH, then treated in the same way as GA samples,. More liquefying buffer was added when necessary. To ensure the accuracy of the test results, the CSF samples should not be less than 0.5ml, and the thick specimens should be fully digested.

#### DNA extraction and CPA testing

**Figure 1:**
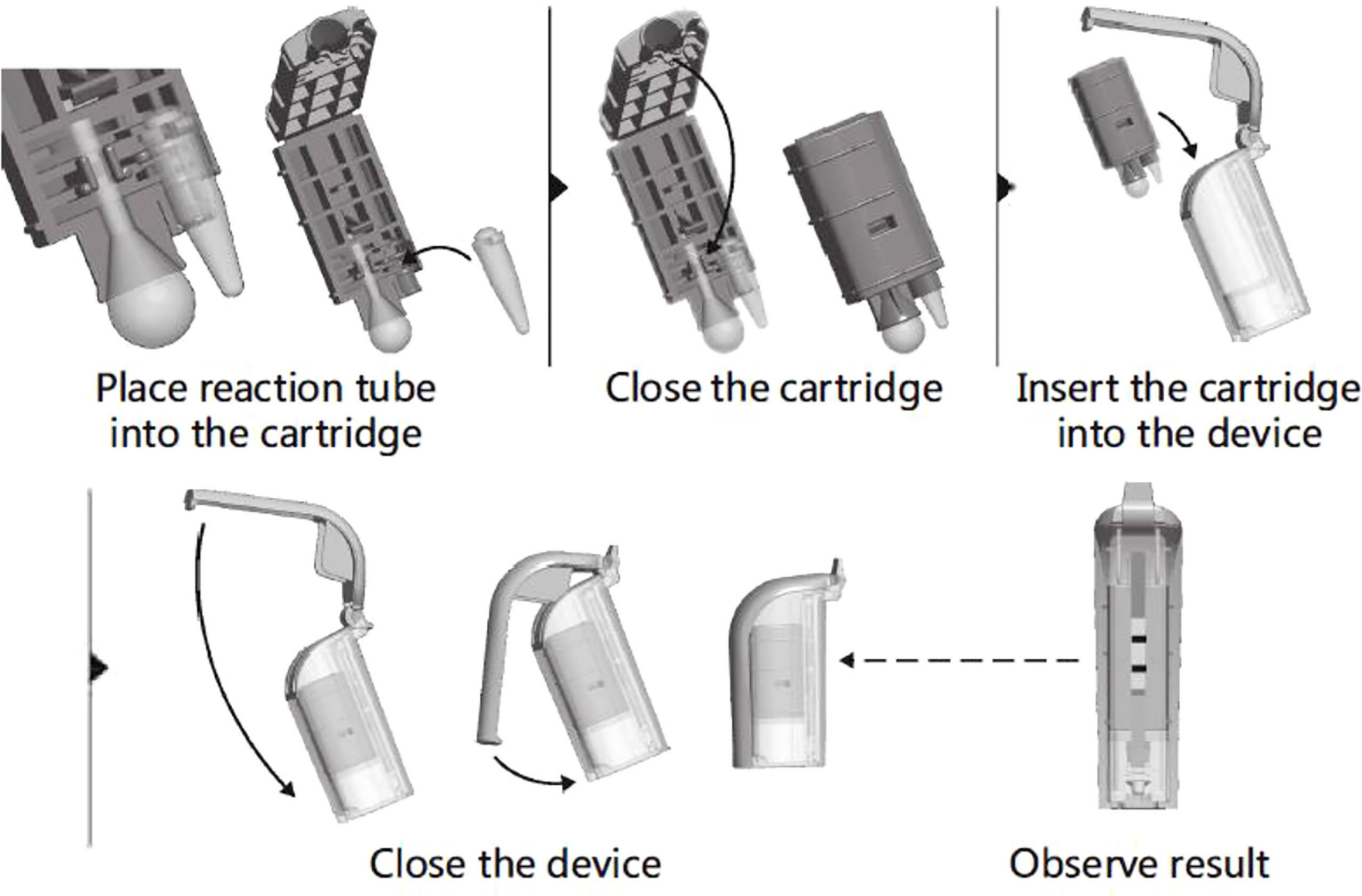
Aperation of the disposable Nucleid Acid Detection Device.

All the operators from participating units were trained on the CPA(Ustar Biotechnology Co., Ltd, Hangzhou, China) test from November to December 2018, and all study samples were handled strictly according to the operating instructions. Liquified samples (1 mL) were centrifuged at 10,000 rpm for 10 min in a 1.5-ml centrifuge tube, and the precipitate was washed twice with 1 mL physiological saline (0.9% w/v NaCl) and centrifugation at 10,000 rpm for 10 min. The pellet was resuspended in 40 μL of DNA Extraction Solution and incubated at 100°C for 10 min. Tubes were cooled to room temperature and centrifuged at 10,000 rpm for 5 min, then the supernatant was collected as the amplification template.The DNA template (4 μL) was added to a reaction tube containing vitrified reagent and resuspending buffer, then covered with paraffin oil. Positive and negative controls were set with the positive control reagent and ddH_2_O that came with the kit. All reaction tubes were briefly centrifuged for 3~5 s at 4,000 rpm before being placed on a 63°C heater for a 60-min amplification reaction. The target gene is IS6110 of MTBC. A folded cartridge containing the tube was placed in the disposable Nucleic Acid Detection Device and locked in position (**Figure 1**). Each CPA assy result was determined by observing visible bands on the test strips.

### Xpert MTB/RIF assay

The specimen was mixed with the standard treatment solution at a ratio of 1:2 (if the specimen was thick, the ratio was ≥1:3; CSF or relatively clear serous cavity effusion was not processed for direct detection), then vortexed for 15~30 s and allowed to stand at room temperature for 15 min. The mixed solution (2.1~2.5 mL) was transferred into a disposable multi-chamber plastic reaction box and placed in the GeneXpert detection system (Cepheid, USA) for automatic detection.

### Interpretation of results and data collection

To avoid false positive detections, CPA positive samples were also confirmed by Culture or Xpert MTB/RIF or Fluorescence quantitative PCR or CPA test again or next generation sequencing, according to the different laboratory conditions of the participating units.

Data were saved in an electronic medical records system (hospitalization and clinic visits) and data analysis was anonymous. Demographic information, clinical manifestations, tuberculosis exposure history, BCG vaccination status, clinical symptoms, laboratory findings were recorded.

### Statistical analysis

The data were analyzed with SPSS 23.0 software (SPSS Inc., Chicago, USA) using the chi-square test or Fisher’s exact test, as appropriate. A *P-*value of < 0.05 was considered to indicate a significant difference.

## Results

### Assessment of CPA assay for tuberculosis diagnosis in children

To first evaluate the accuracy of the CPA assay for diagnosis of childhood tuberculosis (TB), data from 50 children with culture-positive active tuberculosis (ATB) and 109 non-TB children (159 children in total) were analyzed. The non-TB cases were typically diagnosed as having mycoplasma pneumonia, bronchiectasis, lung edema, and other pulmonary illnesses. The sensitivity was 96% (48/50) by the CPA method and 98% (49/50) by Xpert MTB/RIF, and the specificity of both methods were 100% (109/109) compared to culture method. SPSS26.0 was used to analyze the agreement and response correlation between CPA and Xpert MTB/RIF in 159 subjects. Kappa index was used to measure concordance between the assays. In the 159 subjects, CPA and Xpert MTB/RIF were both positive in 47 and both negative in 109 subjects, with an almost perfect agreement(κ= 0.96, 95% CI: 0.85–0.95, *P < 0.001*; **Table 1**).

**Table 1:** Agreement between CPA and Xpert MTB/RIF.

Xpert MTB/RIF	CPA			Kappa value (%)
	+	-	Total	
**+**	47	2	49	0.96
**-**	1	109	110
Total	48	111	159	

Based on the above results, we established that the CPA method is reliable for the diagnosis of tuberculosis in children.

### Characteristics and the MTBC positive rates distribution of all subjects

A total of 851 cases were enrolled, and all subjects’ characteristics are shown in **Table 2**. 58.9% (501/851) of subjects were male, the MTBC positive rate in males was 8.6% (43/501), and there was no significant difference with females (10.9%, 38/350) (*P*=0.957). The median age of the enrolled patients was five years, ranging from 1 day to 18 years old. The majority of enrolled patients were under the age of 6 (preschool period). The MTBC positive rates in 12-18-year-old group (middle school) was 26.4% (24/91), higher than that in the < 6-year-old (preschool period) and the 6~11-year-old groups (primary school) combined (*P* < 0.001).

**Table 2:** The clinical characteristics of MTBC-positive subjects.

Groups	MTBC positive rates in total,%	*P*	MTBC positive rates in cases with suspected PTB, %	*P*	MTBC positive rates in cases with suspected EPTB, %	*P*
Gender						
male	8.6 (43/501)	0.957	10.4 (29/280)	0.099	6.3 (14/221)	0.59
female	10.9 (38/350)		15.0 (31/206)		4.9 (7/144)	
Age, years						
< 6†	7.4 (39/525)	<0.001	9.0 (23/255)	<0.001	5.9 (16/270)	0.498*
6~11	7.6 (18/235)		9.4 (15/160)		4.0 (3/75)	
12~18	26.4 (24/91)		31 (22/71)		10.0 (2/20)	
TB contact history						
Yes	35.3 (18/51)	<0.001*	36.8 (14/38)	<0.001*	30.8 (4/13)	0.004*
No	7.8 (61/782)		10.1 (44/435)		4.9 (17/347)	
Uncertain	11.1 (2/18)		15.4 (2/13)		0 (0/5)	
BCG vaccination						
Yes	9.5 (8/846)	/	12.4 (60/483)	/	5.8 (21/363)	/
No	0 (0/5)		0 (0/3)		0 (0/2)	
With Congenital immunodeficiency †						
Yes	33.3 (7/21)	0.01*	0 (0/5)	/	43.8 (7/16)	<0.001*
No	8.39 (74/830)		12.5 (60/481)		4.0 (14/349)	

*Fisher’s exact test. † Among 21children with congenital immunodeficiency, 8 were less than 1 year old and 6 of them had BCGosis.

Only 5 patients (0.6%) did not receive BCG vaccination. 51 cases(6.0%)had a clear TB contact history and 782 cases (91.9%) had no history of exposure to tuberculosis. The MTBC positive rate(35.3%, 18/51) in patients with a clear history of tuberculosis exposure was significantly higher than in cases that had no TB contact history (*P* < 0.001).

The MTBC-positive rate of patients with congenital immune deficiency was 33.3% (7/21; **Table 2**), higher than that of patients without congenital immune deficiency (*P*=0.01*). Among the 7 patients, 4 cases were due to abscess ulceration at the BCG vaccination site between 6 months and 18 months old after BCG vaccination, and 2 cases of lymphoid tuberculosis had contact history with tuberculosis patients and developed the disease at 1 month and 8 months, respectively. The cause of infection was not established in 1 child with abdominal tuberculosis.

As a supplement, among the 49 samples tested positive by XpertMTB/RIF, 9 samples (18.4%, 9/49) were tested resistance against RIF.

### Suspected PTB cases: Characteristics and MTBC-positivity

Pulmonary TB (PTB) was suspected in 486 (57.1%) cases. From them, 520 specimens were collected, including 290 bronchoalveolar fluid samples, 87 gastric fluid samples, 76 sputum samples, and 67 pleural puncture fluid samples. 60 of the suspected PTB cases were confirmed MTBC-positive (12.3%).

As shown in **Table 2**, there was no significant difference in the positivity rate of MTBC between males and females (*P* = 0.099), the patients aged from 12 to 18 years old had higher infection rates (31.0%, 22/71) than those in the other groups (*P < 0.001*). The rate of MTBC positivity (36.8%, 14/38) was significantly higher in patients with a clear history of tuberculosis exposure than in other groups (*P < 0.001*).

### Suspected EPTB cases: Characteristics and MTBC-positivity

**Figure 2:**
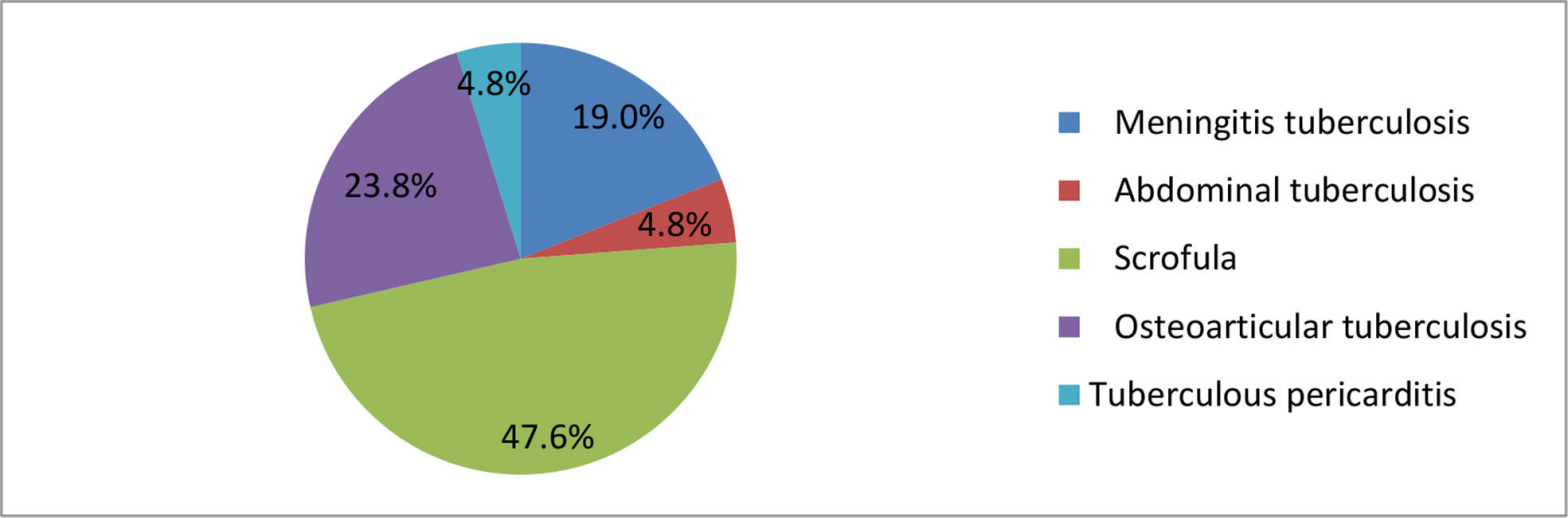
Proportions of EPTB types.

Of the 365 cases with suspected EPTB, 21 (5.8%) were MTBC positive (**Table 2**). Ten (47.6%) of these cases were scrofula, among them eight of the patients who were less than one year old were found to have BCG disease, and all of them were born with an immune deficiency disorder (**Table 2**). 5 (23.8%) cases were osteoarticular tuberculosis, 4 (19%) cases were meningeal tuberculosis, and single cases of an abdominal and a pericardial tuberculosis were found (**Figure 2**).

There was no significant difference in the EPTB positivity rate between males and females, or between age groups. Similar to the PTB group, known tuberculosis exposure was associated with MTBC-positivity (30.8%, 4/14; *P* = 0.004). In addition, we found that the MTBC positive rate in patients with proven congenital immunodeficiency (43.8%) was higher than that in patients without congenital immune deficiency (*P* < 0.001).

### The geographical distribution of the cases of childhood TB

**Figure 3:**
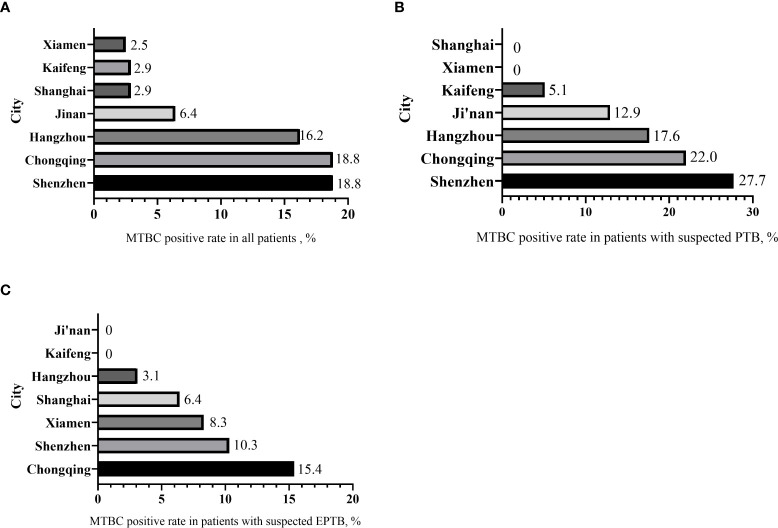
The distribution of MTBC-positive rates by city. **(A)** MTBC positive rate in all patients. **(B)** MTBC positive rate in patients with suspected PTB. **(C)** MTBC positive rate in patients with suspected EPTB.

Among the 851 subjects enrolled, the geographical distributions were: 125 (14.7%) from Jinan, 105 (12.3%) from Kaifeng, 175 (20.6%) from Shanghai, 117 (13.7%) from Chongqing, 74 (8.7%) from Hangzhou, 122 (14.3%) from Xiamen and 133 (15.6%) from Shenzhen. The overall MTBC-positve rate was 9.5%. The positive rates of MTBC for enrolled patients in different regions were: 6.4% (8/125) in Jinan, 2.9% (3/105) in Kaifeng, 2.9% (5/175) in Shanghai, 18.8%(25/133) in Chongqing, 16.2% (12/74) in Hangzhou, 2.5% (3/122) in Xiamen, and 18.8% (25/133) in Shenzhen respectively (**Figure 3A**).

In patients with suspected PTB, the MTBC-positive rates were: 27.7% (18/65) in Shenzhen, 22.0% (20/91) in Chongqing, and 17.6% (11/42) in Hangzhou, 12.9% (8/62) in Jinan, and 5.1% (3/59) in Kaifeng. No positive cases were found in Shanghai and Xiamen (**Figure 3B**).

In patients with suspected EPTB (**Figure 3C**), Chongqing had the highest positive rate 15.4% (4/26), followed by Shenzhen 10.3% (7/68), and Xiamen 8.3% (3/36), Shanghai 6.4% (6/94), Hangzhou 3.1% (1/32). There were no positive cases in Jinan and Kaifeng.

### The results from the different kinds of clinical specimens

In total, 917 specimens from all participants were collected for analysis. The most common sample type was bronchial lavage fluid specimens (BL; 32.5%, 298/917), followed by cerebrospinal fluid specimens (CSF; 27.8%, 255/917), puncture fluid specimens (PF; 21.3%, 195/917), gastric aspirate specimens (GA; 9.4%, 86/917), and sputum specimens (SP; 9.1%, 83/917). The positive rates of MTBC in different specimens are shown in **Table 3**.

**Table 3:** The positive rate of MTBC in different specimens.

Specimen	Total number	Total		< 6 years-of-age		6~18 years-of-age	
		Number of positives	%	Number of positives	%	Number of positives	%
GA	86	18	20.9	10	11.6	8	9.3
SP	83	8	9.6	2	2.4	6	7.2
BL	298	32	10.7	10	3.4	22	7.4
CSF	255	4	1.6	3	1.2	1	0.4
PF	195	22	11.3	13	6.7	9	4.6
Total	917	84	9.2	38	4.1	46	5.0

### Clinical symptoms associated with a confirmatory diagnosis of MTBC

Of the 81 MTBC-positive patients, 60 were diagnosed with pulmonary tuberculosis (PTB) and 21 were diagnosed with extrapulmonary tuberculosis (EPTB). For patients with PTB, the clinical presentations were fever (51.7%), cough for more than two weeks (48.3%), dyspnea (13.3%), weight loss (6.7%), abdominal pain (5.0%), vomiting (5.0%), night sweats (3.3%), disturbance of consciousness (3.3%). Patients with EPTB commonly presented with night sweats (33.3%), fever (28.6%), arthralgia (19%), cough for more than 2 weeks (14.3%), and vomiting (9.5%) (**Table 4**).

**Table 4:** Clinical symptoms of MTBC-positive subjects.

Clinical symptoms	PTB (n = 60)		EPTB (n = 21)	
	Number	Percent	Number	Percent
Fever	31	51.7	6	28.6
Cough > 2 weeks	29	48.3	3	14.3
Dyspnea	8	13.3	0	0
Weight loss	4	6.7	0	0
Abdominal pain	3	5.0	1	4.8
Emesis	3	5.0	2	9.5
Night sweats	2	3.3	7	33.3
Disturbance of consciousness	2	3.3	1	4.8
Lymphadenopathy	1	1.7	0	0
Headache and/or convulsions	1	1.7	1	4.8
Arthralgia	0	0	4	19
Initially symptomatic, symptoms found during physical examination	6	10	0	0

## Discussion

Molecular methods are recommended for TB diagnosis by WHO and are used in many countries. EasyNAT Diagnostic Kit for Mycobacterium Tuberculosis Complex DNA was developed by Ustar Biotechnologies (Hangzhou) Ltd, China, as an alternative to Xpert MTB/RIF. Compared with the culture method, CPA assay is faster, the results can be reported in about 2 hours. In addition, the results can be found in read in a closed device to avoid contamination.

China, as a high TB burden country with a high incidence of TB in many poor areas, primary laboratories should be equipped with TB screening capabilities, which are particularly important for early diagnosis and treatment of TB and termination of TB. However, many primary hospitals are not equipped to perform MTBC culture and traditional PCR testing, relying only on the Ziehl-Neelsen staining method, which can miss detection easily. The CPA assay is simple, does not require the purchase of expensive instruments, and can be performed by general laboratories at an affordable price, making it ideal for primary laboratories to perform initial screening for TB.

This study is designed mainly based on cross-priming isothermal amplification (CPA) technology. In this study, although only 159 children were tested by both the CPA and Xpert MTB/RIF methods (**Table 1**), we found that the agreement between CPA and Xpert MTB/RIF methods is close to perfect (Kappa value 0.96). Furthermore, the sensitivity and specificity of the CPA test for the various sample types were very high in comparison with the “gold standard” culture method. Hence, we concur that the CPA test is suitable for use in clinical practice for the diagnosis of TB in children, especially in countries with a high TB burden (Wang et al., 2018; Zhang et al., 2021; Quan et al., 2022).

BCGosis can present a diagnostic complication. BCG is an active attenuated *Mycobacterium bovis* tuberculosis vaccine, which can prevent tuberculous meningitis and disseminated tuberculosis infection in infants, and is extensively used in China. The vast majority of infants have no adverse reaction after BCG inoculation, but a small number can develop BCG infection, spread infection, and even cause death in severe cases. According to the detection and analysis of abnormal reactions to suspected BCG vaccination in Shanghai from 2007 to 2017, the reported incidence of BCG abnormal reactions was 10.89 per 100,000 doses, mainly lymphadenitis, while the reported incidence of systemic disseminated BCG disease was 0.21/100,000 doses. In our study, of the 851 children suspected to have MTBC disease, 8 (0.95%) children less than one-year-old were found with BCG disease, and all of them were born with an immune deficiency disorder. Therefore, in regions where the BCG vaccine is administered at birth, clinicians and obstetricians should pay close attention to the possibility of primary immune diseases and BCGosis.

Susceptibility to tuberculosis varies with age. Children are vulnerable to MTBC when they are very young (0~5 years old). However, children aged 5~10 have the lowest infection rate, then the incidence increases during adolescence. (Reyes et al., 2020; Soriano-Arandes et al., 2020) According to China National Tuberculosis Programme (2010-2017), of 40,561 children with tuberculosis, 77.7% (n = 31,529) were aged 10-14 years but only 19.6% (n = 7931) were bacteriologically confirmed, (Li et al., 2020) far from the rates needed for the goal of ending tuberculosis. We found here that among all 851 suspected childhood cases, only 81 (9.5%) cases tested positive (**Table 2**). The difference from the national program may reflect a combination of improved TB control and some misdiagnosis in the national program. Of the cases found here, 60 (7.1%) cases were PTB and 21 (2.5%) were EPTB, EPTB accouts for around 25.9% of the cases. Among EPTB patients, 47.6% (10/21) cases were scrofula, 23.8% (5/21) cases were osteoarticular tuberculosis and 76.2% (16/21) were younger than six years old, consistent with the national findings (Le Roux et al., 2005; Pang et al., 2019).

The patients aged from 12 to 18 years old suffered from higher pulmonary infection rates (31.0%, 22/71) than those in the other groups. The higher rates were probably due to increased interaction with adults when children get in middle school, which raises the risk of exposure to patients with open tuberculosis, and their samples are easy to obtain and contain more bacteria. (Soriano-Arandes et al., 2020) Living conditions including poor housing, poverty, and urban environments are also associated with higher tuberculosis prevalence and transmission. (Stosic et al., 2019; Bekken et al., 2020; Boukamel et al., 2020; Martinez et al., 2020) Unlike adult tuberculosis, there is no gender difference in childhood tuberculosis, (Marais et al., 2004; Nelson and Wells, 2004; Swaminathan and Rekha, 2010) and in this study we did not find gender differences in either PTB or EPTB.

In conclusion, this multicenter investigation confirmed that PTB and EPTB are not uncommon in children in China, teenagers are more susceptible to infection, and granulomatous inflammation of the lymph nodes is the predominant type of extrapulmonary tuberculosis. In contrast to traditional methods, molecular techniques are more sensitive, specific, and less time-consuming, and should be widely used for rapid diagnosis of childhood tuberculosis. The new CPA test is highly suited for use in countries with a high tuberculosis burden.

Particular limitations of this study: most participants had only one specimen tested; social impact factors on childhood tuberculosis remain to be explored.

## Data availability statement

The raw data supporting the conclusions of this article will be made available by the authors, without undue reservation.

## Ethics statement

The studies involving human participants were reviewed and approved by Children’s Hospital of Fudan University. Written informed consent from the participants’ legal guardian/next of kin was not required to participate in this study in accordance with the national legislation and the institutional requirements.

## Author contributions

CL, SW contributed equally to this study, as co-first author. They both analyzed the data and wrote the paper. Author order was determined by contributions to the study. HY, JW, JD, HW, CH, ZZ, LC, JH, WG, HZ, TZ helped with sample collection and testing. Correspondence: CW and HX. All authors contributed to the article and approved the submitted version.

## Acknowledgments

We thank all the technicians and hospitals participating in this investigation for their contributions. We thank Ustar Biotech (Hangzhou, China) for the CPA equipment and detection reagents provided for this project. Thanks to the Key Development Program of Children’s Hospital of Fudan University (EK2022ZX05) Fund for supporting this study.

## Conflict of interest

The authors declare that the research was conducted in the absence of any commercial or financial relationships that could be construed as a potential conflict of interest.

## Publisher’s note

All claims expressed in this article are solely those of the authors and do not necessarily represent those of their affiliated organizations, or those of the publisher, the editors and the reviewers. Any product that may be evaluated in this article, or claim that may be made by its manufacturer, is not guaranteed or endorsed by the publisher.
